# Cross-sectional evaluation of anthropometric measures vs MRI-measured visceral and hepatic fat in a Canadian cohort

**DOI:** 10.1210/jendso/bvag197

**Published:** 2026-09-02

**Authors:** Baraa K Al-Khazraji, Russell J de Souza, Dipika Desai, Jean-Pierre Després, Trevor J B Dummer, Celine Funk, Vikki Ho, Scott A Lear, Jonathon Leipsic, Alan R Moody, Michael D Noseworthy, Grace E Parraga, Paul Poirier, Karleen M Schulze, Jean-Claude Tardif, Jennifer E Vena, Salim Yusuf, Sonia S Anand, the Canadian Alliance for Healthy Hearts and Minds Study Investigators, S Anand, M G Friedrich, D S Lee, P Awadalla, T Dummer, J Vena, G Lettre, V Ho, J Hicks, J-C Tardif, K Teo, S Yusuf, B-M Knoppers, D Desai, S Zafar, K Schulze, S Bangdiwala, C Ramasundarahettige, K Balasubramanian, D Desai, N Tusevljak, K McDonald, N Noisel, J Chu, J Hicks, H Whelan, S Rangarajan, D Busseuil, J Leipsic, S Lear, V de Jong, M Noseworthy, K Teo, E Ramezani, N Konyer, P Poirier, A-S Bourlaud, E Larose, K Bibeau, J Leipsic, S Lear, V de Jong, E Smith, R Frayne, A Charlton, R Sekhon, A Moody, V Thayalasuthan, A Kripalani, G Leung, M Noseworthy, S Anand, R de Souza, N Konyer, S Zafar, G Paraga, L Reid, A Dick, F Ahmad, D Kelton, H Shah, F Marcotte, H Poiffaut, M Friedrich, J Lebel, E Larose, K Bibeau, L Parker, D Thompson, J Hicks, J-C Tardif, H Poiffaut, J Tu, K Chan, A Moody, V Thayalasuthan, M G Friedrich, E Smith, C McCreary, S E Black, C Scott, S Batool, F Gao, A Moody, V Thayalasuthan, E Larose, K Bibeau, F Marcotte, F Henriques, T Teixeira, R de Souza, S Anand, G Booth, J Brook, D Corsi, L Gauvin, S Lear, F Razak, S V Subramanian, J Tu, Jean Rouleau, Pierre Boyle, Caroline Wong, Eldon Smith

**Affiliations:** Department of Kinesiology, Faculty of Science, McMaster University, Hamilton, ON L8S 4K1, Canada; Mary Heersink School of Global Health and Social Medicine, McMaster University, Hamilton, ON L8S 4K1, Canada; Department of Health Research Methods, Evidence, and Impact, Faculty of Health Sciences, McMaster University, Hamilton, ON L8S 4K1, Canada; Population Health Research Institute, Hamilton Health Sciences Corporation, Hamilton, ON L8L 2X2, Canada; Mary Heersink School of Global Health and Social Medicine, McMaster University, Hamilton, ON L8S 4K1, Canada; Department of Health Research Methods, Evidence, and Impact, Faculty of Health Sciences, McMaster University, Hamilton, ON L8S 4K1, Canada; Population Health Research Institute, Hamilton Health Sciences Corporation, Hamilton, ON L8L 2X2, Canada; VITAM – Centre de Recherche en Santé Durable, Université Laval, Québec, QC G1J 2G1, Canada; Centre Intégré Universitaire de Santé et Services Sociaux de la Capitale-Nationale, Québec, QC G1C 3S2, Canada; Département de Kinésiologie, Faculté de Médecine, Université Laval, Québec, QC G1J 2G1, Canada; School of Population and Public Health, University of British Columbia, Vancouver, BC V6T 1Z3, Canada; Population Health Research Institute, Hamilton Health Sciences Corporation, Hamilton, ON L8L 2X2, Canada; Department of Medicine, McMaster University, Hamilton, ON L8S 4K1, Canada; Health Innovation and Evaluation Hub, Centre de recherche du Centre hospitalier de l'Université de Montréal (CRCHUM), Montréal, QC H2X 0A9, Canada; Department of Social and Preventive Medicine, Université de Montréal, Montréal, QC H3T 1A8, Canada; Faculty of Health Sciences, Simon Fraser University, Burnaby, BC V5A 1S6, Canada; Department of Radiology, University of British Columbia, Vancouver, BC V6T 1Z4, Canada; Department of Medical Imaging, Temerty Faculty of Medicine, University of Toronto, Toronto, ON M5S 1A8, Canada; Electrical and Computer Engineering, and Department of Medical Imaging, McMaster University, Hamilton, ON L8S 4K1, Canada; Robarts Research Institute, The University of Western Ontario, London, ON N6A 5K8, Canada; Department of Medical Biophysics, The University of Western Ontario, London, ON N6A 3K7, Canada; Faculté de Pharmacie, Université Laval, Québec, QC G1V 0A6, Canada; Centre de Recherche de l’Institut Universitaire de Cardiologie et de Pneumologie de Québec (IUCPQ), Université Laval, Québec, QC G1V 4G5, Canada; Population Health Research Institute, Hamilton Health Sciences Corporation, Hamilton, ON L8L 2X2, Canada; Department of Medicine, McMaster University, Hamilton, ON L8S 4K1, Canada; Montreal Heart Institute, Montréal, QC H1T 1C8, Canada; Alberta's Tomorrow Project, Cancer Research & Analytics, Cancer Care Alberta, Alberta Health Services, Calgary, AB T2E 7E7, Canada; Population Health Research Institute, Hamilton Health Sciences Corporation, Hamilton, ON L8L 2X2, Canada; Mary Heersink School of Global Health and Social Medicine, McMaster University, Hamilton, ON L8S 4K1, Canada; Department of Health Research Methods, Evidence, and Impact, Faculty of Health Sciences, McMaster University, Hamilton, ON L8S 4K1, Canada; Population Health Research Institute, Hamilton Health Sciences Corporation, Hamilton, ON L8L 2X2, Canada; Department of Medicine, McMaster University, Hamilton, ON L8S 4K1, Canada

**Keywords:** waist circumference, body-mass index, percent body fat, visceral adipose tissue, hepatic fat fraction

## Abstract

**Context:**

Accurate assessment of excess body fat and its cardiometabolic risk is essential in clinical and epidemiologic research. Body-mass index (BMI), although widely used, does not capture visceral or ectopic fat.

**Objective:**

To evaluate how anthropometric measures—BMI, percent body fat, waist circumference (WC), and waist-to-hip ratio (WHR)—relate to MRI-measured visceral adipose tissue (VAT) and hepatic fat fraction (HFF), overall, and by sex.

**Design:**

Cross-sectional analysis within the Canadian Alliance for Healthy Heart and Minds cohort.

**Setting:**

Community-based, pan-Canadian prospective study.

**Patients or Other Participants:**

6683 apparently healthy adults (mean age 57 ± 9 years; 3665 females) with baseline anthropometric and MRI measures; analyses adjusted for study center.

**Intervention(s):**

Not applicable.

**Main Outcome Measure(s):**

MRI-derived VAT and HFF. Associations were estimated using linear regression and mixed models, stratified by sex, ethnicity, age, and BMI category.

**Results:**

Mean BMI was 26.7 kg/m^2^, WC 88.3 cm, VAT 71 mL, and HFF 5.7%. Females had lower VAT and HFF than males. Correlations with VAT ranged from 0.35 to 0.77 and with HFF from 0.26 to 0.47. Each 10 cm higher WC was associated with 21.0 mL higher VAT and 2.1% higher HFF; each 5 kg/m^2^ higher BMI predicted 24.7 mL and 2.8% higher values. When modeled jointly, WC remained strongly predictive, while BMI contributed modestly. Associations were smaller in females. VAT and HFF increased across WC tertiles within BMI categories.

**Conclusion:**

WC is a robust surrogate for visceral and hepatic fat across BMI categories, supporting its use when MRI is not feasible.

Body-mass index (BMI) is widely used to classify individuals as underweight, healthy weight, overweight, or obese; however, its limitations as a measure of excess body fat and associated cardiometabolic risk are well recognized (1-3). BMI may overestimate adiposity in individuals with greater lean mass and underestimate it in older adults or those with reduced muscle mass (4).

Recent expert recommendations have emphasized that BMI should be used primarily as a screening tool and that individual assessment of obesity should incorporate measures of body fat distribution, such as waist circumference (WC) or waist-to-hip ratio (WHR), because BMI alone may not accurately reflect excess adiposity or obesity-related health risk at the individual level (5). Therefore, alternative measures of adiposity, such as WHR, or simply WC have been proposed, which demonstrate stronger associations than BMI with cardiometabolic outcomes including type 2 diabetes and myocardial infarction (1, 6-9).

The distribution of body fat influences its impact on cardiometabolic risk. Visceral adipose tissue (VAT) and hepatic fat fraction (HFF) are of particular concern. Expansion of VAT is a driver of ectopic fat deposition in organs such as the heart, liver, kidneys, and skeletal muscle (10). Hepatic fat accumulation disrupts glucose and lipid metabolism and is closely linked to metabolic dysfunction (11). Although VAT and HFF can be quantified with high precision using magnetic resonance imaging (MRI), these methods are costly, resource-intensive, and not readily scalable for routine clinical use or large population studies.

Several studies have examined the relationship between anthropometric measures and directly measured visceral adiposity. Seminal work from the early 1990s demonstrated that WC and abdominal sagittal diameter were more strongly associated with VAT and related cardiometabolic risk factors than WHR, establishing WC as a practical marker of central adiposity (12). Early MRI- and CT-based investigations subsequently demonstrated that anthropometric measures explain only a modest proportion of the variance of VAT and generally predict subcutaneous adipose tissue more accurately than VAT (13-15). These studies also highlighted important sex differences in the relationship between anthropometric measures and visceral fat accumulation. Later work demonstrated that WC provides information beyond BMI alone, identifying substantial variation in VAT among individuals with similar BMI values (16, 17). Large imaging studies and a meta-analysis of 29 studies including more than 16 000 participants confirmed that WC is the strongest anthropometric correlate of VAT, although substantial unexplained heterogeneity remains (18, 19).

Comparatively fewer studies have examined the correlation of anthropometric measures with hepatic fat accumulation. A recent systematic review and meta-analysis of 42 studies involving more than 42 000 participants reported that BMI and WC were strongly correlated with imaging-derived abdominal and visceral fat, but only weakly to moderately correlated with hepatic fat, suggesting that anthropometric measures may perform differently across ectopic fat depots (20). Measures of abdominal adiposity are associated with hepatic steatosis and metabolic dysfunction-associated steatotic liver disease (MASLD) in studies that have generally relied on CT-derived liver attenuation or clinical definitions of MASLD rather than quantitative MRI-based assessments of liver fat. However, most prior studies have evaluated VAT and hepatic fat separately rather than examining how anthropometric measures relate to both depots within the same population. Consequently, the extent to which commonly used anthropometric measures capture both visceral and hepatic adiposity remains uncertain.

As a result, there is a need for feasible and valid surrogate markers that approximate VAT and HFF in real-world settings. Anthropometric measures, including BMI, percent body fat, WC, and WHR, are widely available and inexpensive, but their relative ability to capture variation in both visceral and hepatic fat across the spectrum of adiposity remains unclear (21, 22). Given that hepatic fat can vary substantially among individuals with similar levels of visceral adiposity (23, 24), surrogate measures that distinguish these phenotypes may improve cardiometabolic risk assessment.

The aim of this analysis is to evaluate how anthropometric measures of general adiposity (BMI and percent body fat) and central adiposity (WC and WHR) relate to both VAT and HFF, as quantified by abdominal MRI, in a large cohort of middle-aged men and women.

## Materials and methods

### Cohort

The present study uses data collected as part of the Canadian Alliance for Healthy Heart and Minds (CAHHM) study, a pan-Canadian cohort established to investigate associations between socio-environmental factors and cardiovascular risk, subclinical CVD, and related chronic conditions (25). Participants were recruited from multiple population-based cohorts across Canada and underwent standardized clinical assessments and MRI imaging at participating centers. Participants completed health questionnaires, physical measurements, and blood sampling, and underwent comprehensive MRI imaging of the brain, heart, carotid arteries, and abdomen (25). A total of 8258 participants were recruited, with stratification to ensure <20% had known CVD, 55% were female, and 20% self-identified as non-white. For this analysis, we included individuals without cancer or chronic obstructive pulmonary disease who provided complete anthropometric data (WC, WHR, and BMI) and MRI-derived VAT or HFF. All participants provided written informed consent, and ethics approval was obtained from the Hamilton Integrated Research Ethics Board (HiREB #13-255) and collaborating sites. This study adheres to STROBE guidelines for reporting observational research (26).

### Data collection

#### Physical measures

Height, weight, percent body fat, WC, and hip circumference (HC) were measured using a standardized protocol by trained study personnel (25, 27). Height was measured barefoot to the nearest 0.1 cm with participants standing erect in the Frankfort plane. Weight and percent body fat were measured with participants wearing light clothing and no shoes using a calibrated foot-to-foot bioelectrical impedance device (Tanita Ironman Innerscan Bc-554). WC was measured at the point of minimal abdominal circumference between the costal margin and iliac crest at the end of a normal expiration, and HC was measured at the level of the greater trochanters (maximum buttock circumference). WC and HC were recorded to the nearest 0.1 cm using a nonstretchable tape measure. WHR was calculated as WC divided by HC.

#### MRI of VAT and HFF

The MRI protocol has been described previously (25, 28, 29). Briefly, participants underwent a short noncontrast scan using a 1.5 or 3.0 Tesla magnet. All 11 local and 1 mobile center completed quality assurance test scans, validated by 4 core MRI labs. VAT was assessed using T1-weighted turbo spin echo (also known as a fast-spin echo) axial sequences at the L4-L5 level, providing a bright fat signal. VAT volumes were analyzed and reported by the core labs. The HFF (%) was measured using dual- and triple-echo gradient-recalled echo sequences from a standardized abdominal protocol on a 3T system with a body coil. HFF values were analyzed and reported by the core lab.

### Statistical analyses

Pearson correlations and scatterplots depict associations of general (BMI and percent body fat) and central adiposity measures (waist and WHR) with VAT and HFF, stratified by sex. Differences in VAT or HFF per specified increment in anthropometric measures were estimated using linear mixed models, with study center included as a random intercept to account for clustering by site and potential variation related to MRI scanner characteristics (eg, manufacturer and magnet strength) across the 10 participating centers. Analyses were further stratified by sex and BMI category. Models including both BMI and WC were compared with models including BMI alone using likelihood ratio tests. Analyses were conducted using SAS version 9.4 (SAS Institute, Cary, NC).

## Results

A total of 6683 participants (average age: 57 ± 9 years, age range: 35 to 69 years) (Table 1) are included in the current analysis (N = 3665 females, N = 3018 males). The mean BMI is 26.7 kg/m^2^ (range: 14.6 to 54.7); the mean % body fat is 30.4% (range: 3.9 to 71.7); the mean WC is 88.3 cm (range: 52.3 to 158.0); and the mean WHR is 0.87 (range: 0.62 to 1.22). For the MRI measures, the mean VAT is 71 mL (range: 8 to 303), and the mean HFF is 5.7% (range: 0.1 to 40.3). Adiposity measures by sex across age and obesity groups are presented in Table 2. Among females, VAT is 61 mL (30) and the HFF is 5.0% (5.4) and among males, VAT is 83 (39) mL and the HFF is 6.5 (6.0) %.

**Table 1:** Demographics and lifestyle characteristics

	N	Overall	Women	Men
Number of participants	6683	6683	3665	3018
Age, mean (SD), y	6683	57.1 (8.8)	56.7 (8.6)	57.7 (8.9)
**Self-reported ethnicity**				
East and Southeast Asian	6683	928 (13.9)	523 (14.3)	405 (13.4)
South Asian	6683	281 (4.2)	122 (3.3)	159 (5.3)
White European	6683	5335 (79.8)	2935 (80.1)	2400 (79.5)
Other^a^	6683	139 (2.1)	85 (2.3)	54 (1.8)
**Highest education attained**				
High school or less	6531	854 (13.1)	512 (14.3)	342 (11.6)
College or trade	6531	2088 (32.0)	1210 (33.8)	878 (29.8)
University Degree	6531	3589 (55.0)	1859 (51.9)	1730 (58.6)
**Smoking status**				
Current	6671	351 (5.3)	180 (4.9)	171 (5.7)
Former	6671	2209 (33.1)	1191 (32.5)	1018 (33.8)
Never	6671	4111 (61.6)	2288 (62.5)	1823 (60.5)
**Health history**				
Diabetes on treatment	6681	307 (4.6)	122 (3.3)	185 (6.1)
Elevated cholesterol %	6683	2405 (36.0)	1062 (29.0)	1343 (44.5)
Hypertension %	6681	2479 (37.1)	1060 (28.9)	1419 (47.0)
Blood pressure, mmHg				
Systolic, mean (SD)	6683	129 (17)	125 (17)	134 (16)
Diastolic, mean (SD)	6683	80 (10)	78 (10)	82 (10)

Presented data are n (%) unless otherwise specified.

^a^Includes Black people, Indigenous, Mixed, and unknown ethnicity.

**Table 2:** Adiposity measures by sex across age groups

		Age categories				
	Females	30-44	45-54	55-64	65+	*P*-trend
Number	3665	331	1131	1449	754	
VAT, mL	61 (30)	45 (24)	56 (28)	64 (30)	70 (30)	<.0001
HFF, %	5.0 (5.4)	3.7 (4.1)	4.6 (5.5)	5.3 (5.8)	5.4 (5.2)	<.0001
**Clinical measures**						
Body fat, %	35.1 (8.1)	33.2 (8.3)	34.6 (8.1)	35.4 (8.0)	36.3 (7.8)	<.0001
BMI, kg/m^2^	26.4 (5.4)	26.0 (5.5)	26.4 (5.5)	26.4 (5.3)	26.6 (5.3)	.08
BMI categories, n (%)						
Normal (<25)	1720 (46.9)	174 (52.6)	540 (47.7)	692 (47.8)	314 (41.6)	.01
Overweight (25-<30)	1146 (31.3)	86 (26.0)	340 (30.1)	449 (31.0)	271 (35.9)	
Obese (30+)	799 (21.8)	71 (21.5)	251 (22.2)	308 (21.3)	169 (22.4)	
WC, cm	84 (14)	82 (14)	83 (14)	84 (13)	86 (14)	<.0001
HC, cm	101 (12)	101 (13)	102 (12)	101 (12)	102 (12)	.33
Waist-to-hip ratio (WHR)	0.83 (0.07)	0.81 (0.07)	0.82 (0.07)	0.83 (0.07)	0.84 (0.07)	<.0001
Abdominal obesity (waist-sex specific), n (%)	1213 (33.1)	94 (28.4)	351 (31.0)	475 (32.8)	293 (38.9)	<.001

		Age categories				
	Males	30-44	45-54	55-64	65+	*P*-trend
Number	3018	246	880	1092	800	
VAT, mL	83 (39)	63 (31)	77 (35)	86 (41)	92 (41)	<.0001
HFF, %	6.5 (6.0)	6.3 (6.3)	6.9 (6.2)	6.5 (6.1)	6.0 (5.3)	.35
**Clinical measures**						
Body fat, %	24.7 (6.8)	22.6 (6.9)	24.1 (6.6)	24.8 (6.8)	25.7 (6.6)	<.0001
BMI, kg/m^2^	27.2 (4.1)	27.1 (4.3)	27.3 (4.2)	27.3 (4.1)	27.1 (4.1)	.99
BMI categories, n (%)						
Normal (<25)	970 (32.1)	94 (38.2)	269 (30.6)	346 (31.7)	261 (32.6)	0.38
Overweight (25-<30)	1409 (46.7)	99 (40.2)	420 (47.7)	514 (47.1)	376 (47.0)	
Obese (30+)	639 (21.2)	53 (21.5)	191 (21.7)	232 (21.2)	163 (20.4)	
WC, cm	94 (12)	91 (12)	93 (12)	94 (12)	96 (12)	<.0001
HC, cm	101 (8)	101 (9)	102 (9)	101 (8)	101 (8)	.97
Waist-to-hip ratio (WHR)	0.93 (0.07)	0.90 (0.06)	0.92 (0.06)	0.93 (0.07)	0.94 (0.07)	<.0001
Abdominal obesity (waist-sex specific), n (%)	673 (22.3)	41 (16.7)	182 (20.7)	231 (21.2)	219 (27.4)	<.0001

Presented data are means (SD) unless otherwise indicated.

*P*-trend calculated using linear contrasts for continuous measures, Cochran Armitage Test for categorical variables with 2 levels, and CMH Test for categorical variables with >2 levels.

### Associations of anthropometric measures with VAT

Correlations between each of these measures and VAT range from 0.35 to 0.77 (Fig. 1A-1D). In models adjusted only for a random effect of center, increases in BMI (per 5 kg/m^2^), % body fat (per 5%), WC (per 10 cm) and WHR (per 0.05 unit) corresponded to 24.7 mL (95% CI: 24.0 to 25.4), 6.4 mL (95% CI: 6.0 to 6.9), 21.0 mL (95% CI: 20.6 to 21.4) and 13.7 mL (95% CI: 13.3 to 14.1) higher VAT, respectively. When BMI and WC are included in the same model, WC still predicts practically the same volume of VAT (19.6 mL per 10 cm; *P* < 0.0001), whereas BMI contributes only modestly (2.2 mL per 5 kg/m^2^; *P* < 0.0001). Adding WC to a model with BMI explains more variability than BMI alone (LRT statistic = 2217.2; *P* < 0.001) but adding BMI to a model with WC adds comparatively less value (LRT statistic = 18.1; *P* < 0.001).

**Figure 1:**
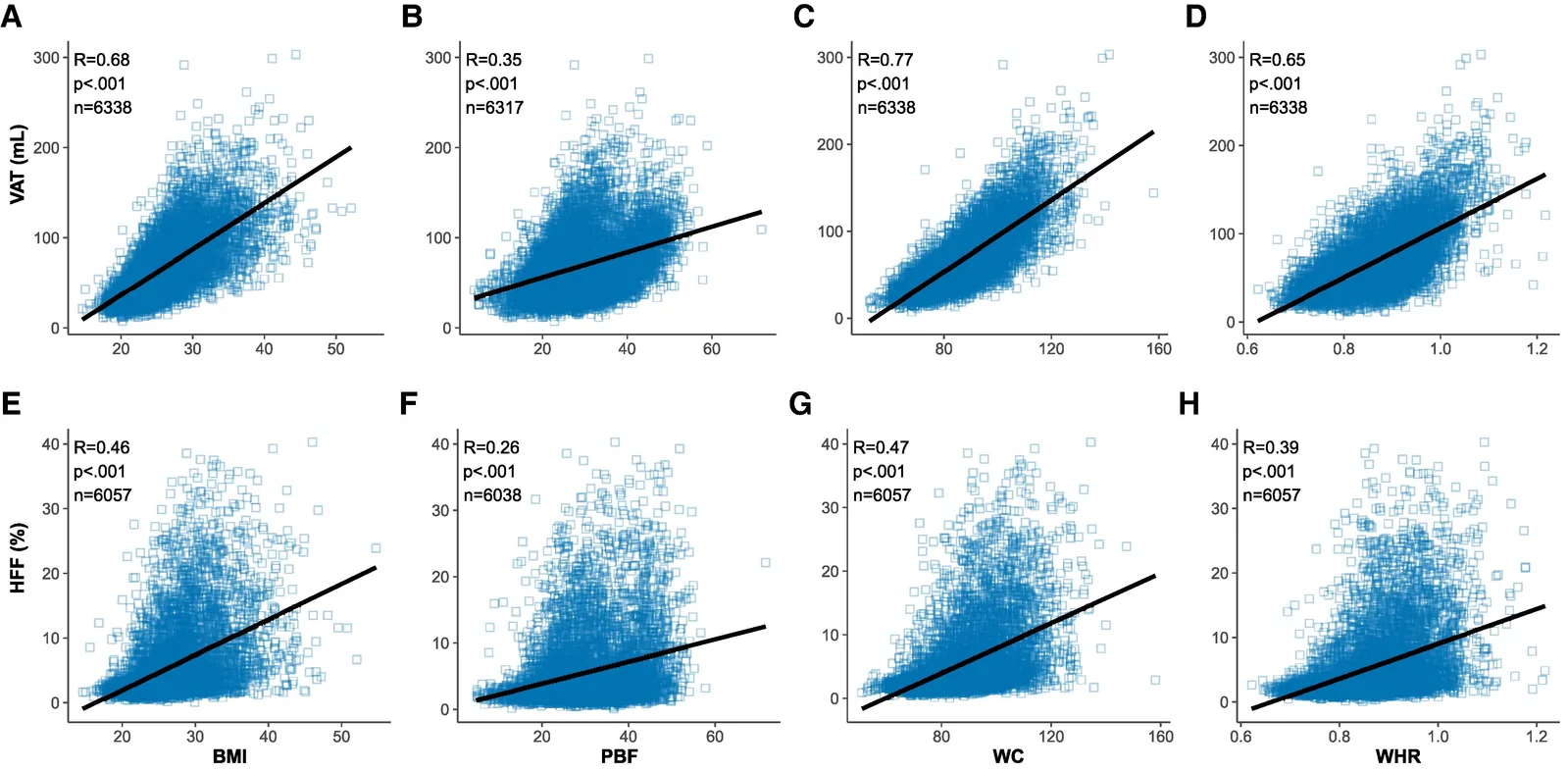
Correlations of anthropometric measures with visceral adipose tissue (VAT) volume and hepatic fat fraction (HFF). Scatterplots show the associations of body mass index (BMI; panels A and E), body fat percentage (PBF; panels B and F), waist circumference (WC; panels C and G), and waist-to-hip ratio (WHR; panels D and H) with VAT volume (panels A-D) and HFF (panels E-H). Pearson correlation coefficients (R), *P*-values, and sample sizes are displayed within each panel. Solid lines represent fitted regression lines. All associations were positive and statistically significant (*P* < 0.001).

### Sex-specific associations of anthropometric measures with VAT

Correlations between measures range from 0.54 to 0.77 in females and 0.67 to 0.75 in males (Fig. 2A-2D). For the same unit increase in BMI, percent body fat, WC, or WHR, VAT increases are smaller in females than males. In both sexes, WC is the most informative predictor when modeled with BMI. In females, each 10 cm higher WC predicts 14.4 mL higher VAT vs 5.0 mL for a 5-unit higher BMI (*P* < 0.0001). In males, each 10 cm higher WC predicts a 23.6 mL higher VAT volume vs 3.3 mL for a 5-unit higher BMI (*P* < 0.0001) (Fig. 3).

**Figure 2:**
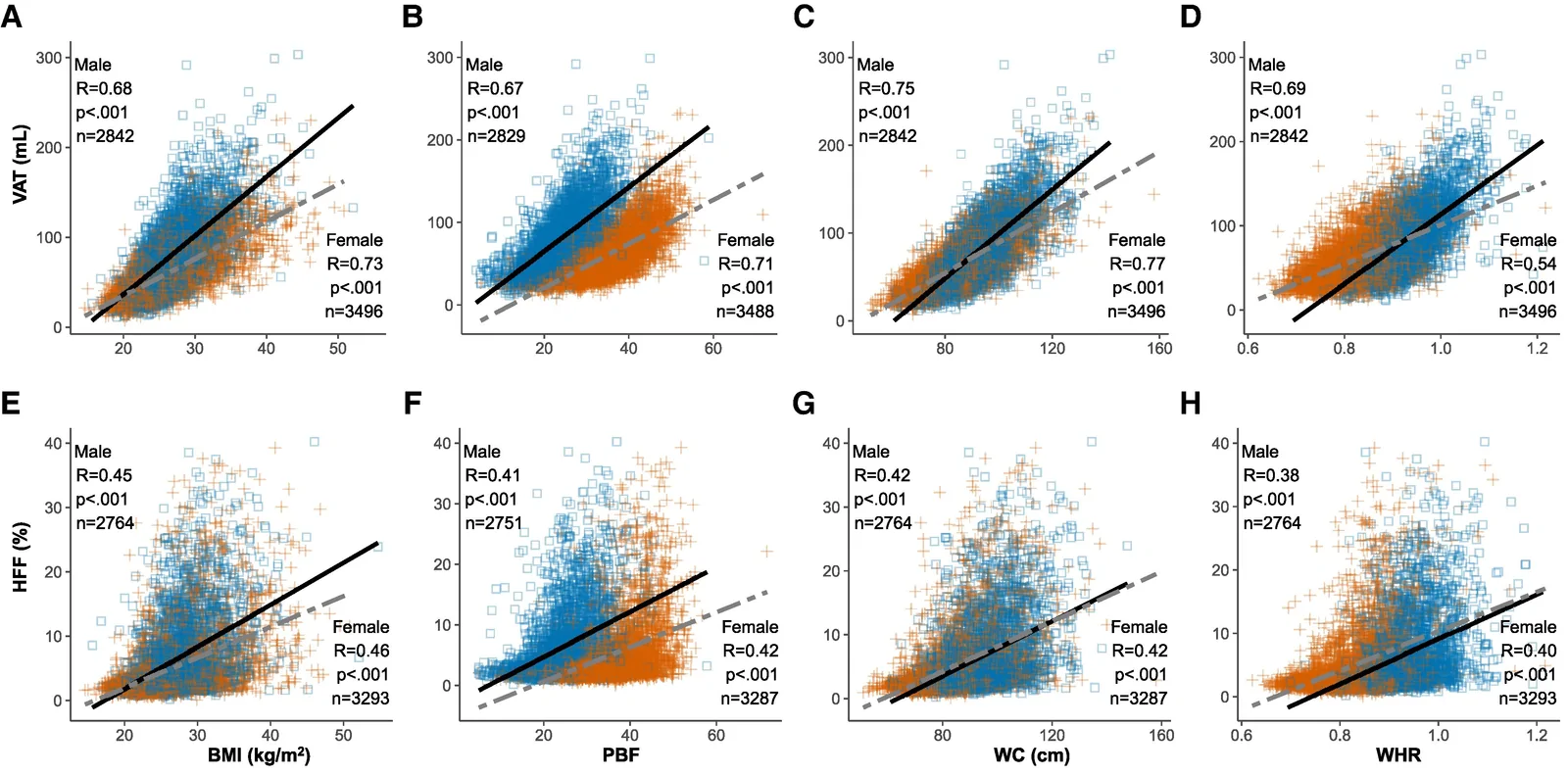
Scatterplots show the associations of body mass index (BMI), percent body fat (PBF), waist circumference (WC), and waist-to-hip ratio (WHR) with VAT volume (panels A-D) and HFF (panels E-H) among males and females. Correlation coefficients (R), *P*-values, and sample sizes are presented within each panel. Solid and dashed lines represent sex-specific fitted regression lines. All correlations were statistically significant (*P* < 0.001).

**Figure 3:**
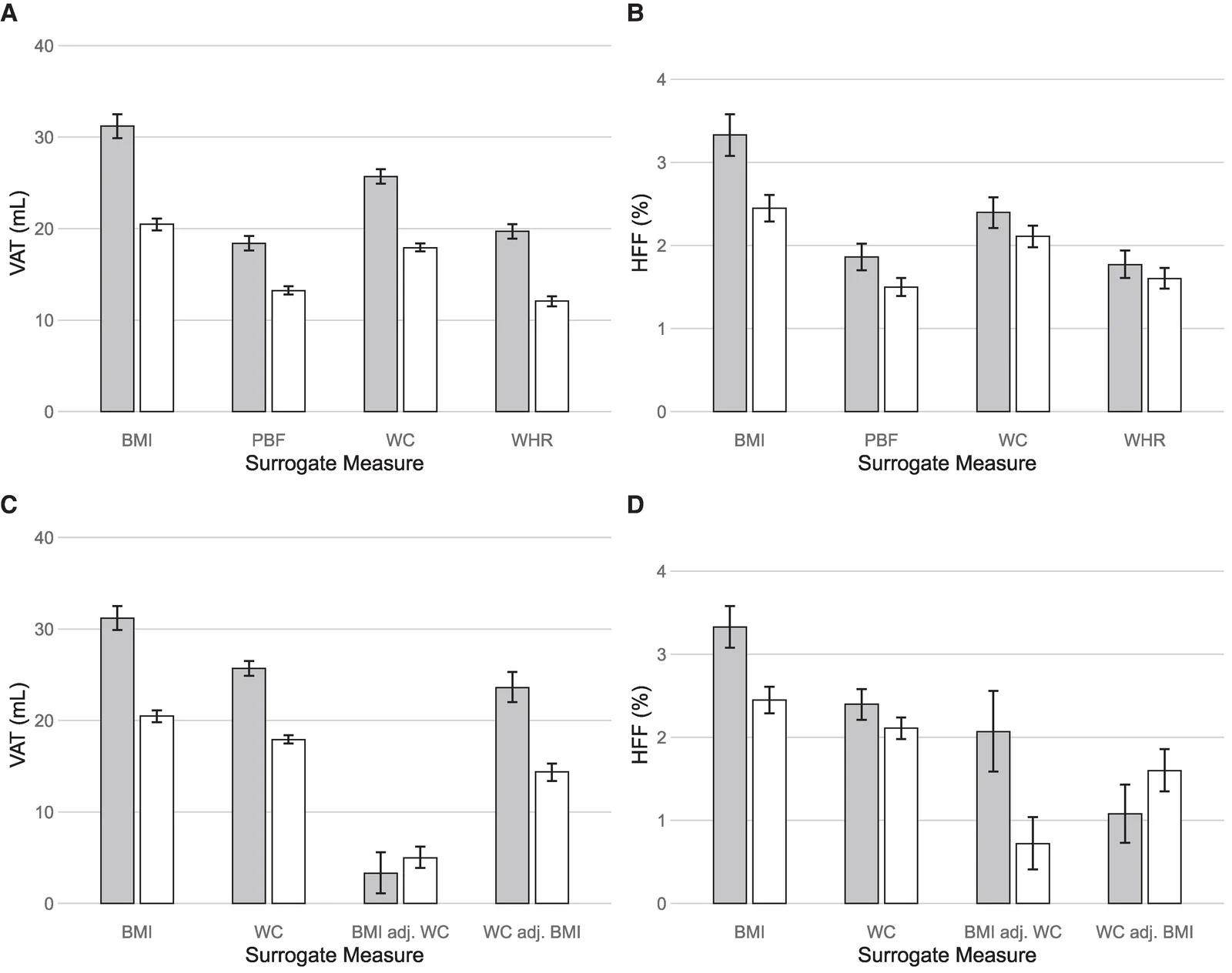
Associations of anthropometric measures with VAT and HFF. Bars represent the estimated increase in VAT (mL) or HFF (%) associated with a specified increment in each anthropometric measure obtained from linear mixed models, with 95% confidence intervals shown by error bars. Top panels: (A) Estimated increase in VAT associated with a 5 kg/m² higher BMI, 5% higher body fat percentage (%BF), 10 cm higher WC, or 0.05-unit higher WHR, shown separately for males and females. (B) Estimated increase in HFF associated with the same increments in BMI, %BF, WC, and WHR. For all anthropometric measures, the corresponding increases in VAT and HFF were significantly greater in males than females (all tests for sex interaction *P* < 0.001). Bottom panels: (C) Estimated increase in VAT associated with a 5 kg/m² higher BMI (unadjusted model), a 10 cm higher WC (unadjusted model), BMI after additional adjustment for WC, and WC after additional adjustment for BMI. (D) Estimated increase in HFF associated with the same models. These analyses illustrate the independent contributions of BMI and WC when included simultaneously in the model.

### Associations of anthropometric measures with HFF (%)

Correlations between measures and HFF ranged from 0.26 to 0.47 (Fig. 1E-1H). In models adjusted only for a random effect of center, increases in BMI (per 5 kg/m^2^), % body fat (per 5%), WC (per 10 cm) and WHR (per 0.05 unit) corresponded to 2.8% (95% CI: 2.7 to 2.9), 0.84% (95% CI: 0.77 to 0.92), 2.1% (95% CI: 2.0 to 2.6) and 1.35% (95% CI: 1.27 to 1.43) higher HFF, respectively. When BMI and WC are included in the same model WC remains strongly predictive of HFF (1.4% higher per 10 cm; *P* < 0.0001), while BMI shows a smaller estimated effect (1.2% higher HFF; *P* < 0.0001). Adding WC to a model with BMI explains more variability than BMI alone (LRT statistic = 240.9; *P* < 0.001) but adding BMI to a model with WC adds comparatively less value (LRT statistic = 91.5; *P* < 0.001). When WC and BMI are included together, WC remains strongly predictive of HFF (1.4% higher per 10 cm; *P* < 0.0001), while BMI shows a smaller estimated effect (1.2% higher HFF; *P* < 0.0001).

### Sex-specific associations of anthropometric measures with HFF%

Correlations range from 0.40 to 0.46 in females and 0.38 to 0.45 in males (Fig. 2E-2H). In females, WC is the stronger predictor (1.6% per 10 cm vs. 0.7% per 5 kg/m^2^), whereas in males, BMI is stronger (2.1% vs. 1.1%; *P* < 0.0001 for all) (Fig. 3).

### Associations of anthropometric measures with VAT and HFF by BMI category

VAT increased progressively across WC tertiles within each BMI category and was consistently higher in males compared to females (Fig. 4). Among participants with BMI < 25 kg/m^2^, VAT ranged from 43 to 67 mm^3^ in males and from 37 to 50 mm^3^ in females. For BMI 25-<30 kg/m^2^, VAT increased from 69 to 101 mm^3^ in males and from 54 to 75 mm^3^ in females. At BMI ≥ 30 kg/m^2^, VAT ranged from 99 to 144 mm^3^ in males and from 80 to 111 mm^3^ in females. The sex difference in VAT was greater in magnitude with increasing BMI, from ∼15 cm^3^ at BMI < 25 kg/m^2^ to over 30 cm^3^ at BMI ≥ 30 kg/m^2^.

**Figure 4:**
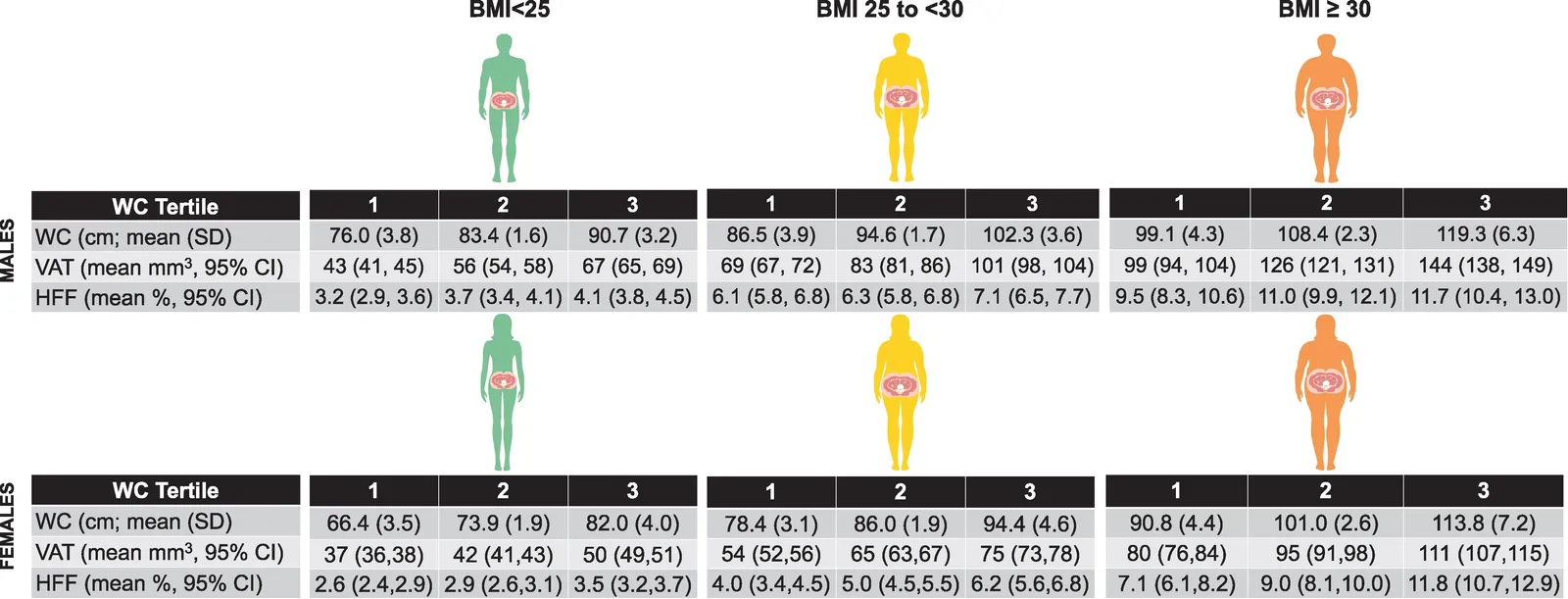
BMI-adjusted mean values and 95% CI of VAT and HFF by within-BMI-category tertile of WC. Mean values of VAT (mm^3^) and HFF (%) consistently increase across tertile of WC (cm) within each BMI category, in both males (top) and females (bottom). BMI categories are: normal weight; <25 kg/m^2^; overweight 25 to <30 kg/m^2^; and obese ≥30 kg/m^2^ (n = 6338). *P*-trend across WC tertile within BMI categories are: <0.0001 for VAT in both males and females; and 0.001, 0.02, and 0.02 for HFF in males; and <0.000 for HFF in females. This figure incorporates Shutterstock images ID 716496511 (MarShot), 688185661 (MarShot), and 18709773496 (matsukiyo8379), licensed by Russell de Souza under a Shutterstock Standard License purchased on November 12, 2025. The image is reproduced in accordance with the terms of the Shutterstock license.

HFF increased across WC tertiles within each BMI category and was consistently higher in males compared to females (Fig. 4). Among participants with BMI < 25 kg/m^2^, HFF ranged from 3.2% to 4.1% in males and from 2.6% to 3.5% in females. For BMI 25-<30 kg/m^2^, HFF increased from 6.1% to 7.1% in males and from 4.0% to 6.2% in females. At BMI ≥ 30 kg/m^2^, HFF ranged from 9.5% to 11.7% in males and from 7.1% to 11.8% in females. While males generally exhibited higher HFF values at lower BMI categories, the sex difference was less pronounced at BMI ≥ 30 kg/m^2^, where females in the highest WC tertile had slightly higher HFF than males.

## Discussion

This analysis investigates how clinically feasible anthropometric measures relate to VAT and HFF in a large Canadian cohort of adult men and women. WC emerged as the strongest predictor of VAT and HFF compared with BMI. For the same unit-increases in these measures, females consistently had lower VAT and HFF than males, reflecting sex differences in fat distribution. Within BMI categories, higher WC predicted greater VAT and HFF, even after accounting for BMI variability. These findings emphasize the importance of considering sex and BMI category when interpreting anthropometric data. Importantly, VAT and HFF are potent predictors of incident diabetes (30-35), cardiovascular events (36), and heart failure (37, 38), underscoring the clinical relevance of identifying individuals with excess visceral and hepatic fat early using anthropometric tools.

This study confirms that WC is more strongly associated with VAT and HFF than BMI, even in sex-stratified analyses. This supports prior evidence that WC is the best single measure for identifying cardiovascular risk factors (12, 39). WC primarily reflects visceral/ectopic and subcutaneous fat around the waist. HC, used in the WHR, reflects both adipose tissue and lower-body muscle mass, making WHR harder to interpret. A high WHR may indicate excess abdominal fat or low gluteofemoral muscle, while a low WHR can occur in obesity with large hips, leading to misclassification. HC is also harder to measure clinically and adds little value to CV risk assessment beyond WC. Given rising cardiometabolic disease in diverse, aging populations, WC offers an inexpensive, and scalable approach to risk identification, notably in settings where MRI or CT capacity is limited.

Our findings support the growing view that BMI and measures of central adiposity provide complementary rather than competing information (5, 40). BMI remains a practical and useful marker of overall adiposity and an effective population-level screening tool, while WC provides additional information about visceral fat distribution and related cardiometabolic risk. However, individuals with similar BMI values can differ substantially in the amount of visceral and ectopic fat they carry, and these depots appear more closely linked to cardiometabolic risk. In this context, WC may be particularly valuable because it captures variation in VAT that is not fully reflected by BMI alone. These observations are consistent with recent recommendations that assessment of obesity should extend beyond BMI and incorporate measures of body fat distribution when feasible. Rather than replacing BMI, routine measurement of WC may improve identification of individuals with excess visceral adiposity who could otherwise be overlooked using BMI alone.

WC offers several advantages for clinicians and public health professionals. It is inexpensive, noninvasive, and can be obtained within minutes using minimal equipment, making it feasible for implementation in primary care and community-based settings. Broader adoption would require standardized measurement protocols, training of clinical staff, and incorporation of WC into routine clinical documentation. Nevertheless, because advanced imaging methods such as MRI are not scalable for routine screening, WC may provide a pragmatic means of improving identification of individuals with excess visceral adiposity in a wide range of health-care settings.

VAT and HFF are individually associated with cardiometabolic risk. The recent KORA-MRI study (N = 299) using whole-body MRI found the largest effect sizes for VAT in relation to hypertension, and for HFF in relation to diabetes outcomes when comparing adipose tissue relationships with cardiometabolic risk factors (41). Similarly, the Framingham Heart Study reported that higher VAT predicts incident CVD and cancer, independent of other risk factors and general adiposity (42). In CAHHM, we have recently shown higher VAT and to a lesser extent, HFF, are associated with greater carotid atherosclerosis (29). Despite these strong associations, gold-standard imaging methods such as MRI and CT remain costly, time-intensive, and inaccessible in many clinical settings, limiting their scalability. These constraints reinforce the utility of WC as a practical surrogate for identifying high-risk fat distribution. The Interheart case-control study showed WHR to be superior to BMI for predicting myocardial infarction (43). Given the global rise in cardiometabolic disease, WC offers an inexpensive, and equitable approach for early risk stratification where advanced imaging is not feasible.

Including WC and BMI in the same model provides more information than BMI alone. In our models, WC predicts VAT and HFF more strongly than BMI, consistent with prior evidence that the effect of BMI is attenuated (and often, inverted) when WC is included (44, 45). When WC is included in the same model, BMI primarily reflects lean mass and height. These findings align with studies showing WC combined with BMI predicts CV risk factors better than BMI alone (46, 47). The graded increase in predicted VAT and HFF with higher WC, but not BMI, across BMI categories shows the added value of WC for identifying individuals at particularly high cardiovascular risk due to excess visceral and hepatic fat, even among those with generalized overweight or obesity (48). These findings reinforce the concept that incorporating WC into routine assessment may refine cardiometabolic risk stratification beyond BMI alone, enabling earlier identification of individuals with high visceral or hepatic fat despite being classified within the same BMI category.

Associations between anthropometric measures and VAT and HFF vary by age and sex. Men typically have higher VAT and HFF levels than women, while VAT and HFF change across the lifespan in women but remain stable in men. These patterns likely reflect hormonal influences. Perimenopausal women store fat in gluteofemoral regions due to estrogen receptor activity, whereas declining estrogen after menopause promotes abdominal fat redistribution (49-52). Women also show lower HFF than men across most age groups except in older age, consistent with higher risk for MASLD in men earlier in life, and increased risk in women postmenopause (53-55). Recognizing these sex-specific trajectories is essential for tailoring preventive strategies, particularly as women experience accelerated visceral fat accumulation during the menopausal transition.

### Strengths and limitations

The strengths of our study include our large sample size, gold-standard measures of both VAT and HFF via MRI acquisition, and analysis of a diverse ethnic cohort of otherwise healthy Canadians. Although we suggest that the differential changes in VAT volume between males and females are attributed to hormonal shifts with menopause, the hormonal profiles were not assessed, and self-reported menopause data was not concurrent with the MRI data for most of the study population. A limitation of BIA is that the foot-to-foot device does not measure fat through the abdomen, unlike MRI which assessed only abdominal regions, and its estimates of body fat percentage can vary with hydration status, which was not measured.

## Conclusion

In summary, WC is an informative, accessible, and clinically relevant anthropometric measure for identifying individuals with excess visceral and hepatic fat. Given the strong links between VAT, HFF, and cardiometabolic disease, adding WC alongside BMI to routine clinical assessments and population health screening could substantially improve early detection of high-risk individuals, particularly in settings where MRI is not feasible.

## Data Availability

CAHHM data cannot be deposited publicly as these collaborative data originate from multiple Canadian cohorts with different legal frameworks. CAHHM represents a “cohort of cohorts,” and at the time participants were enrolled into each respective cohort, data sharing was not part of the consent process. Thus, the appropriate legal frameworks to allow for data to be deposited publicly (identified or de-identified) is not in place, nor was such sharing anticipated or run by the respective REB at the time of cohort invitations. Therefore, any requests for data may be made to CAHHM (Alliance@phri.ca), with requests for data sharing to be considered on a case-by-case basis. A detailed statistical analysis plan and the code used in the analysis are also available on reasonable request.
